# Applications for Bacteriophage Therapy during Pregnancy and the Perinatal Period

**DOI:** 10.3389/fmicb.2017.02660

**Published:** 2018-01-11

**Authors:** Lucy L. Furfaro, Barbara J. Chang, Matthew S. Payne

**Affiliations:** ^1^Division of Obstetrics and Gynecology, School of Medicine, The University of Western Australia, Crawley, WA, Australia; ^2^Marshall Centre for Infectious Disease Research and Training, School of Biomedical Sciences, The University of Western Australia, Crawley, WA, Australia

**Keywords:** bacteriophages, phage therapy, antenatal, perinatal, pregnancy, fetus, antibiotic prophylaxis, bacterial infections

## Abstract

Pregnant women and their unborn children are a population that is particularly vulnerable to bacterial infection. Physiological changes that occur during pregnancy affect the way women respond to such infections and the options that clinicians have for treatment. Antibiotics are still considered the best option for active infections and a suitable prophylaxis for prevention of potential infections, such as vaginal/rectal *Streptococcus agalactiae* colonization prior to birth. The effect of such antibiotic use on the developing fetus, however, is still largely unknown. Recent research has suggested that the fetal gut microbiota plays a critical role in fetal immunologic programming. Hence, even minor alterations in this microbiota may have potentially significant downstream effects. An ideal antibacterial therapeutic for administration during pregnancy would be one that is highly specific for its target, leaving the surrounding microbiota intact. This review first provides a basic overview of the challenges a clinician faces when administering therapeutics to a pregnant patient and then goes on to explore common bacterial infections in pregnancy, use of antibiotics for treatment/prevention of such infections and the consequences of such treatment for the mother and infant. With this background established, the review then explores the potential for use of bacteriophage (phage) therapy as an alternative to antibiotics during the antenatal period. Many previous reviews have highlighted the revitalization of and potential for phage therapy for treatment of a range of bacterial infections, particularly in the context of the increasing threat of widespread antibiotic resistance. However, information on the potential for the use of phage therapeutics in pregnancy is lacking. This review aims to provide a thorough overview of studies of this nature and discuss the feasibility of bacteriophage use during pregnancy to treat and/or prevent bacterial infections.

## Use of therapeutics in the antenatal period

Pregnancy induces a number of physiological changes in women, ranging from changes in energy metabolism (Herrera, 2000; Prentice and Goldberg, 2000) to maternal circulation (Hunter and Robson, 1992), and presents a number of unknown risks in relation to the pharmacokinetics of different therapeutics (Loebstein et al., 1997). Infection during pregnancy can have devastating outcomes for the mother and fetus and is a major cause of preterm birth (delivery at <37 weeks gestation) (Goldenberg et al., 2008). Minimizing harm to the developing fetus is of the utmost importance when treating maternal conditions during this period, however, the complex nature of pregnancy can make this a challenging task. Maternal treatment can often result in the fetus being unnecessarily treated and conversely, attempts to treat the fetus *in utero* by maternal administration of drugs can vary greatly depending on the placental transfer of the chosen compound (Pacifici, 2006). This is further confounded by the overall lack of knowledge of the interactions of various therapeutics during pregnancy, largely due to this population being excluded from most clinical trials of drug efficacy as a result of safety concerns (Ke et al., 2014).

A review by Anderson examined pharmacokinetic factors during pregnancy to assess the differences in drug absorption, metabolism, distribution and excretion (Anderson, 2006). The issue of drug transfer to the fetus *in utero* is of particular concern, given the poorly understood impacts that exposure may have during these early stages of development. The placenta plays a number of roles during pregnancy: not only does it act as a physical barrier, it also provides exchange of nutrients and oxygen and differentiates the maternal and fetal circulation. Drug transfer during pregnancy may occur across the placenta through passive diffusion or transporter systems (Syme et al., 2004). Improved knowledge of the mechanisms of such transfer and the extent of metabolism prior to contact with the fetus is important in understanding how drug administration during pregnancy may affect the neonate. With this in mind, one would think that drug safety for use in pregnancy would certainly be thoroughly investigated, however, a survey of the global clinical trials registries reveal that only 0.32% of active registered studies were pregnancy drug trials (Scaffidi et al., 2017). As the majority of drugs prescribed and used during pregnancy are off-label and often never formally tested for use in pregnancy (Ke et al., 2014), the exclusion of pregnant women from such trials is ethically questionable considering that the results may be of great relevance to this population; however, extensive consideration of the trial design is required for this population due to the greater inherent risks (Welch et al., 2015). In addition, as highlighted by Welch and colleagues, a depth of understanding must be attained to ensure women are able to make informed decisions about their own treatment and involvement in trials.

## Antibiotics during pregnancy

### Bacterial infections

The majority of antibiotic use during pregnancy is targeted at treating various urinary tract infections (UTIs), sexually transmitted infections (STIs) such as *Treponema pallidum, Neisseria gonorrhoeae* and *Chlamydia trachomatis* and the common food-borne pathogen *Listeria monocytogenes* (Ross, 1979; Brumfitt and Hamilton-Miller, 1981; Cram et al., 2002). Another pathogen associated with neonatal sepsis, *Streptococcus agalactiae* or Group B Streptococcus (GBS), is also targeted with antibiotics in an effort to prevent transmission to the neonate during birth (Verani et al., 2010). These pathogens are targeted in an effort to prevent poor obstetric outcomes, however, even narrow-spectrum antibiotics impact on a number of other bacterial species in addition to the target. Alternatively, broad-spectrum antibiotics are commonly used in the event of complications during pregnancy such as premature rupture of membranes (PROM) (Chapman, 1986). *In utero* infection is a major cause of preterm birth, with preterm births accounting for 11% of all live births (Blencowe et al., 2012), and is often associated with significant morbidity and mortality (Goldenberg et al., 2000). Major causes of infection-associated preterm birth include the *Ureaplasma* spp., for which treatment options are particularly limited due to their lack of a peptidoglycan layer rendering all β-lactam antibiotics ineffective (Viscardi, 2014). All of these bacterial infections and a number of others have resulted in widespread administration of antibiotics to pregnant women, the majority of which have limited data available on their relevant safety profiles and are broad-spectrum in their activity, affecting not only the target organisms, but also commensal bacteria.

### Safety

With the above causes of infection and pharmacological considerations in mind, it has been reported that 80% of all prescriptions during pregnancy are antibiotics and approximately 20-25% of women will receive antibiotics during pregnancy (Heikkila, 1993; Santos et al., 2010; de Jonge et al., 2014; Bookstaver et al., 2015). A review of antibiotic use during pregnancy by Bookstaver et al. (2015) included an extensive list of antibiotics accompanied by their Food and Drug Administration (FDA) pregnancy category rating from A (no risk in pregnancy) to X (contraindicated in pregnancy). The list only contained categories B (generally safe to use) to D (avoid in pregnancy unless benefit outweighs risk). This further highlights the lack of knowledge we have on the effect of drugs, specifically antibiotics, which are widely used during pregnancy. In general β-lactams, macrolides, clindamycin and fosfomycin are considered safe during pregnancy, but additional studies are required for further assessment of other antibiotic classes that currently have minimal pregnancy data (Bookstaver et al., 2015).

In addition to selecting the appropriate antibiotic, the correct dose of antibiotics is essential to minimize toxicity and maximize efficacy. During pregnancy, pharmacokinetic properties of drugs can be expected to vary significantly in comparison with non-pregnant adults due to a number of physiological changes (Pariente et al., 2016). Further to this, the dose a fetus receives from maternal administration significantly differs depending on a number of factors, one being the placental barrier. Drugs must overcome the placental barrier to reach the fetus and have a therapeutic effect; the rate of this transplacental transfer is low for widely-used macrolide antibiotics (Philipson et al., 1973; Heikkinen et al., 2000; Ramsey et al., 2003). Heikkinen and others observed transfer of erythromycin, roxithromycin and azithromycin to be as low as 3, 4.3, and 2.6%, respectively, across a placental perfusion model (Heikkinen et al., 2000). Extensive studies by Kemp and colleagues have examined the *in vivo* effect of erythromycin, azithromycin and more recently, solithromycin, using a pregnant sheep model (Keelan et al., 2011; Kemp et al., 2014a,b). The clearance of *Ureaplasma parvum* intrauterine infection by IV administration was achieved by azithromycin and solithromycin (Miura et al., 2014). In contrast, neither intramuscular nor intra-amniotic administration of erythromycin was able to completely resolve *U. parvum* intra-amniotic infection (Kemp et al., 2014b). This highlights not only the importance of drug bioavailability but also efficacy against the pathogen.

Drug bioavailability to the fetus is important for treating intrauterine infections, while conversely, minimal transfer may be beneficial when treating maternal infection as the lack of transfer results in limited fetal exposure. Philipson and colleagues examined ampicillin (Philipson, 1977), cepharidine and cephazolin (Philipson et al., 1987): both studies concluded that higher doses were required during pregnancy to reach similar plasma levels as compared to after pregnancy. This raises questions regarding under and over dosing in this population; the former has the potential to impact on resistance in bacterial infections as sub-inhibitory doses can select for resistant isolates, and higher dosage raises issues of toxicity (Anderson, 2006; Pariente et al., 2016). Most antibiotics cross the placenta to some extent. β-lactams cross rapidly and equilibrate in maternal and cord plasma in what is termed “complete” transfer, while other antibiotics show “incomplete” transfer where concentrations are lower in the cord than maternal plasma (Pacifici, 2006).

### Antibiotic resistance

Widespread antibiotic use is strongly associated with the development of multi-drug resistant (MDR) bacteria. The World Health Organisation (WHO) recently published a list of priority organisms that require urgent drug development to overcome resistance to current antibiotics (Willyard, 2017). Of the pathogens mentioned, *N. gonorrhoeae* is listed as “High Priority” for research and development of new antibiotics. While resistance is more prevalent in some bacterial species than others, continued widespread use coupled with a lack of new drug development is likely to continue to fuel the growth of MDR strains amongst numerous new species in the coming years (Norrby et al., 2005; Talbot et al., 2006; Ventola, 2015). In fact, some recent predictions suggest total human mortality attributed to MDR bacteria by 2050 could exceed that resulting from cancer (O'Neill, 2014).

### Microbial dysbiosis

The majority of antibiotics have broad spectrum activity and affect not only the target bacteria but also commensal organisms. In addition to potentially promoting resistance amongst organisms other than the intended target/s, commensal disruption also results in a state of antibiotic-induced dysbiosis in which those bacteria unaffected by the antibiotic (potentially already resistant or protected within a microbial biofilm) populate the microbial community through lack of competition (Mendling, 2016). The resulting cascade of impacts possible is far reaching, from adverse effects on various maternal body site microbiomes, through to effects on the seeding of the neonatal microbiome *in utero* and soon after delivery (Mueller et al., 2015; Stinson et al., 2017). The latter in particular is of great significance when considering intrapartum antibiotic prophylaxis (IAP) which aims to delay antibiotic exposure until the point of delivery in an effort to reduce exposure and prevent neonatal infection through vertical transmission, particularly for GBS colonization (Schrag and Verani, 2013). This has successfully reduced incidence of GBS disease in neonates (Verani et al., 2010), however, a number of studies have now assessed the impact of this exposure on neonatal gut microbiomes.

Mazzola and colleagues noted the effect in IAP-exposed breastfed infants following analysis of infant stool samples, where 16S rDNA sequences associated with the *Enterobacteriaceae* were increased at seven days and remained in high abundance a month after delivery (Mazzola et al., 2016). IAP resulted in a reduction in *Bifidobacterium* spp. sequences in breastfed and mixed-fed infants at 7 days, but numbers returned to levels comparable with non-IAP exposed infants by 1 month of age. Similarly, another group examined the effect of IAP for GBS on the gut microbiota within the 1st month of life. This study selected microbial groups to examine including *Lactobacillus* spp., *Bifidobacterium* spp., and *Bacteroides fragilis* (Corvaglia et al., 2016). They observed a reduction in Bifidobacteria counts at 7 days of life in the IAP group compared to no IAP. However, breast milk-fed infants had increased *Lactobacillus* spp. counts regardless of IAP at both 7 and 30 days of life. Another study revealed differences in infant gut microbiota communities at 3 months of age following IAP exposure (Azad et al., 2016). Further disruption was evident in those who received IAP during cesarean section delivery, with taxonomic differences in microbial profiles persisting to 12 months in those cesarean section-delivered, formula-fed infants who received IAP. These studies highlight the potential effects of IAP on the neonatal gut microbiome and suggest that it can be modified and semi-restored through breastfeeding. However, the long-term impact of this initial microbial seeding displacement is not yet well understood (Azad et al., 2016; Corvaglia et al., 2016; Mazzola et al., 2016). Reviews on the significance of the fetal microbiome for infant health highlight the effect such prophylactic treatments have on the initial microbial diversity and the impact this may have on other health parameters (Dunlop et al., 2015; Mueller et al., 2015; Yang et al., 2016; Stinson et al., 2017). In light of this information, when considering IAP for a defined target, such as for the prevention of GBS vaginal/rectal transmission, the potential for use of targeted, non-antibiotic therapies warrants serious consideration.

Without a doubt, antibiotics represent a significant breakthrough in medical history. In an antenatal context they have prevented large amounts of maternal and neonatal morbidity, however, there is limited information available on their safety for use in pregnancy. The development of resistant bacteria, although not a major issue in pregnancy at present, is still a serious concern. Although substantial contention still exists (Perez-Munoz et al., 2017) surrounding recent evidence that the newborn likely develops its initial gut microbiota *in utero* and that this is seeded from the mother (Dunlop et al., 2015; Mueller et al., 2015; Stinson et al., 2017), regardless, it would certainly appear that targeted antimicrobial therapies, such as bacteriophages, would be of significant benefit during the antenatal period. This is particularly so in cases of prophylactic treatment, such as prevention of GBS infection. Broad-spectrum antibiotic use is certainly still warranted though when unknown potential pathogens are involved, in cases of PROM for instance (Seelbach-Goebel, 2013), but reserving usage to these emergency situations would likely be of significant benefit to both the mother and the developing fetus in terms of reducing microbial dysbiosis and potential associated adverse effects on fetal immune programming.

## Bacteriophages

Bacteriophages are bacterial viruses also known as phages. They represent the most abundant organisms on Earth. They are so expansive in number that it has been postulated that there are likely a trillion phages for every single grain of sand on the planet (Keen, 2015). Unlike other viruses, bacteriophages are only able to infect bacteria and remain distinct from animal and plant viruses (Carlton, 1999). The co-discovery of these viruses was made in 1915 by Twort (1915) and independently 2 years later by d'Herelle who subsequently named them bacteriophages (D'Hérelle, 1917). Upon the realization that phages had the capability of killing bacteria, their use as a therapeutic was investigated almost immediately. In the century of research since their discovery, many breakthroughs, especially in molecular biology, have been made through the use of bacteriophages (Keen, 2015). Initial interest in these viruses was, however, overshadowed by the emergence of cheap and effective antibiotics which have since been the frontline in bacterial infection treatment. However, recent years have brought attention to the serious issue of emerging antibiotic resistance, which has revitalized the notion of using bacteriophages as therapeutic agents (Nobrega et al., 2015).

### Mechanism of action

The overall mechanism of action that all phages undertake includes adsorption, injection of genetic material into host, replication, assembly and virion release. Given the breadth of phage taxa there are a number of variations to the following process, however, as an obligate pathogen the phage must find a host in which to replicate. The process of adsorption occurs in two steps, the first is the initial contact with the host surface through reversible electrostatic forces, allowing the phage to survey the host at closer proximity to locate specific receptors. In the case of tailed phages, capsid interaction with the host surface allows the tail components to search for and interact with specific receptors on the surface (Hu et al., 2013; Murata et al., 2017). If the specific host receptor is found, tailed phages will bind irreversibly to their target and complete the adsorption process. Alternatively, tailless and filamentous phages contain the necessary components for attachment at exposed surface sites and have evolved to use host-cell-encoded channels for genomic transfer (Peralta et al., 2013).

Once attached to the host, the genetic material of the phage, initially encased in the phage head or capsid, is injected into the host where it will then undergo replication. Larger phages, such as ϕKZ, contain genomes that encode their own necessary DNA replication requirements, while others may rely on the host (Ceyssens et al., 2014). The replication process involves synthesis of numerous copies of the genetic material, translation and the manufacture of phage components such as the head, tail and internal proteins. Once matured and assembled these virions are released from the host cell via internal lysis, after which the progeny may go on to infect other cells and continue the infection process, whilst the host is killed (Stent, 1963). Other methods of progeny release can also include budding and extrusion (Weinbauer, 2004).

Bacteriophage lifecycles include obligately lytic, lysogenic, pseudolysogenic and chronic infection (Clokie et al., 2011). Bacteriophages are often classified according to the best understood of these lifecycles as virulent (obligately lytic) or temperate (lysogenic lifestyle) (Dy et al., 2014). Recent review of phage terminology has highlighted terms that require classification, such as the intended use of “lytic and lysogenic” and these suggestions have been incorporated into this review (Hobbs and Abedon, 2016). Obligately lytic phages do not integrate with the host and only enter the lytic cycle. This cycle involves cessation of host component production and utilization of host products to replicate phage products, ultimately resulting in host cell death and release of assembled virions (Stent, 1963; Young, 2013; Roach and Donovan, 2015). This feature makes these phages the perfect subjects for bacteriophage therapy as the goal is to eradicate the target bacteria in a bactericidal fashion (Brussow, 2012). The lytic cycle acts to regulate bacterial populations and is beneficial in many ways, such as in the reduction of colonization by pathogens of mucosal surfaces. Barr et al. demonstrated that bacteriophage colonizing the mucosal surfaces of humans and animals limited bacterial populations and acted as an additional component of innate immunity (Barr et al., 2013). Their ubiquitous presence makes it difficult to believe they wouldn't play a significant role in our individual microbiomes and health. Indeed, Manrique and colleagues have demonstrated a shared “phageome” of humans and propose that it plays a major part in maintaining the structure and function of the gut microbiome, although the temperate phages discussed below are believed to be more abundant than obligately lytic phages in the human gut (Manrique et al., 2016).

Temperate phages, however, can undertake a lysogenic lifestyle which involves phage replication that does not directly result in virion production or release. These phages have the ability to integrate into the host genome or exist as a plasmid, enabling replication through host reproduction and resulting in bacterial daughter cells containing this phage genome (prophage). The temperate phage can remain as a prophage, coexisting in the lysogenized host which it leaves unharmed until conditions are unfavorable for growth, in which case the phage, if integrated, may then excise itself from the genome and enter a lytic cycle.

Both obligately lytic, and temperate phages may exhibit pseudolysogeny, a controversial topic but defined by some authors as a state of stalled development of a phage within its host, in which neither phage replication nor prophage formation occurs (Łoś and Wegrzyn, 2012). This state is triggered by conditions that cause sub-optimal growth of bacteria or starvation, allowing the co-existence of both the resulting inactive and unstable phage, and the starved host within low resource environments without mutual destruction (Brendan and Lenski, 1997). Pseudolysogeny is resolved with the change of nutrient conditions which enable growth, at which point the phage can either form a stable prophage or enter the lytic cycle. Chronic phage infection may also involve either temperate or non-temperate phages (Hobbs and Abedon, 2016), and refers to productive infection in which virions are released over time without host cell lysis. Filamentous phages have the ability to release progeny phage through extrusion via membrane transport complexes (Rakonjac et al., 2011).

Lysogeny makes temperate phages a potential public health risk as phage-encoded virulence genes or regulators can result in increased host pathogenesis as a result of phage expression. The process of phage conversion involves the expression of a different host phenotype as a result of temperate phage infection. Phage-encoded virulence factors are numerous, and thus temperate phage infection could result in increased pathogenicity of the lysogenized host bacteria (Clokie et al., 2011; Keen, 2015). Prime examples of phage-mediated virulence exist in *Vibrio cholerae*, in which the gene for production of the cholera toxin is contained on a phage (CTXϕ, an example of a chronic temperate phage) (Waldor and Mekalanos, 1996) and the same is evident in *Escherichia coli* with phage-encoded shiga-like toxin contributing to the symptoms of hemorrhagic colitis (O'Brien et al., 1984). As well as numerous toxin genes, temperate phages encode other traits that promote bacterial colonization, uptake and survival within a host cell (reviewed by Boyd) (Boyd, 2012).

Considering this brief overview of phage biology, we highlight that the obligately lytic phages currently provide the best option for therapeutic use due to their lethal effect on the bacterial host, as opposed to the co-existence with the host of temperate phages and the potential problem of lysogenic conversion.

### Bacteriophage therapy

The lethal nature of obligately lytic bacteriophages is highly attractive for exploitation in treatment of bacterial infections. Certain characteristics of bacteriophages such as the specificity of their action, are a significant advantage over antibiotics when targeted therapy is applicable (Table 1). This specificity can improve treatment outcomes for patients in that only specific bacterial species within an individual host range are removed, leaving other beneficial microbes unaffected (Loc-Carrillo and Abedon, 2011).

**Table 1:** Comparison of therapeutic characteristics of bacteriophages and antibiotics.

**Bacteriophages**	**Antibiotics**
**BACTERICIDAL AGENTS**	
Virulent phages cause cell lysis (bactericidal). Bioengineering of phages can produce bacteriostatic results.	Induce a static state (bacteriostatic) or cause cell death (bactericidal).
**DOSAGE**	
Dose escalation through release of exponential phage numbers caused by host-dependent replication.	Dose is dependent on absorption, concentration, metabolism and excretion of the agent.
**TOXICITY**	
Generally considered as inherently non-toxic due to nucleic acid and protein composition. Risk of lysed cell remnants that may lead to severe reactions in therapeutic use unless phages are highly purified.	Range of toxicity levels which can vary according to the drug depending on contraindications such as pregnancy.
**MICROBIOTA DISRUPTION**	
Host specificity of phages results in minimal to no disruption of the normal microbiota.	Broad-spectrum antibiotics are well known for disrupting the overall microbiota and causing a dysbiotic state. Narrow spectrum antibiotics reduce this impact, but still target many species.
**RESISTANCE**	
The specificity of phages for their hosts means that if resistance does emerge only a select bacterial population will be affected. Phage-resistant mutants are often less virulent as phage receptors are commonly associated with pathogenicity. Cocktail formulations reduce resistance emergence and may be used to treat antibiotic-resistant pathogens.	Broader range of activity means that a substantial proportion of bacterial species are affected and the potential for widespread resistance to emerge is greater. Mutations resulting in resistance to one antibiotic may cause cross-resistance to other agents.
**DISCOVERY**	
Phage discovery is generally rapid and relatively easy due to their ubiquitous nature.	Antibiotic discovery is expensive and complicated by drug design and development in addition to assessment of potential toxicity. Minimal new discoveries.

*Information adapted from Loc-Carrillo and Abedon (2011)*.

## Bacteriophages against antenatal pathogens

### Urinary tract pathogens

Development of urinary tract infections (UTIs) can occur at any stage during pregnancy and often results in antibiotic intervention, which depending on the gestation, could have implications for fetal development (de Tejada, 2014). Numerous *in vitro* studies have outlined the level of activity that isolated bacteriophages have on specific pathogens and a number of cocktails have been developed to cover a broader host range and reduce risk of resistance forming (Lehman and Donlan, 2015; Galtier et al., 2016; Sybesma et al., 2016). Common causes of UTIs, *E. coli* and *Klebsiella pneumoniae* have been the target of a number of studies. Sybesma and others reported the activity of commercially available bacteriophage cocktails from the George Eliava Institute in Georgia against clinical *E. coli* (*n* = 41) and *K. pneumoniae* (*n* = 9) strains isolated from urine of patients with UTIs, where activity against all strains except one was observed (Sybesma et al., 2016). The ability to penetrate biofilms is of particular relevance when considering these as treatment options. One study found a significant reduction of uropathogenic *E. coli* biofilms within 2–12 h of phage administration *in vitro* (Chibeu et al., 2012). Additionally, a continuous-flow *in vitro* model using artificial urine was used to assess the prevention of biofilm formation by pre-treatment of urinary catheters with phages, which showed a significant reduction in common UTI species *Pseudomonas aeruginosa* and *Proteus mirabilis* (Lehman and Donlan, 2015).

The genitourinary tract is the major focus when considering applications for bacteriophage use antenatally, and in the context of UTI treatment during pregnancy the common antibiotic side-effect of vaginal dysbiosis/candidiasis can be prevented with such targeted therapies. In addition, it is well established that opportunistic pathogens residing in the vaginal microbiota during pregnancy can ascend through the cervix and establish florid infections of the fetal membranes (chorioamnionitis), amniotic cavity and ultimately the fetus (Goldenberg et al., 2000). The asymptomatic nature of such vaginal colonization can have devastating impacts on the immune-naïve fetus and neonate and in this context pathogen removal using specific bacteriophages could be implemented as a preventative measure.

*Ureaplasma* spp. are prime examples of such asymptomatic colonization's, with 40 to 80% of pregnant women colonized with these bacteria (Waites et al., 2005). Their role in preterm birth has been one of controversy, however, studies highlighted in the review by Capoccia et al. (2013) address the association between *Ureaplasma* spp. and adverse pregnancy outcomes. In addition, more recently, in a prospective cohort study of low-risk pregnant women, Payne and colleagues reported that it is *U. parvum*, not *Ureaplasma urealyticum*, that is of relevance to preterm birth and particularly *U. parvum* genotype SV6 (Payne et al., 2016). Intra-amniotic infection can lead to preterm birth and additional neonatal morbidity and mortality (van Waarde et al., 1997; Viscardi and Hasday, 2009; Kasper et al., 2011; Kallapur et al., 2013). *Ureaplasma* spp. are a potential target for bacteriophage therapy, however, their unique biological characteristics represent a challenge. They are unable to form a lawn or turbid culture due to their small cell size, making plaque assays and other standard phage techniques extremely difficult.

Galtier and colleagues examined phage therapy for UTIs in the form of uropathogenic *E. coli* eradication from the gut of mice using a cocktail of three virulent bacteriophages (Galtier et al., 2016). In this study they found that a single oral gavage dose of the bacteriophage cocktail was able to remove the target *E. coli* with less effect on microbial diversity than that of antibiotic administration. Another study found that a bacteriophage cocktail was effective against *E. coli* adhered to the urothelium, commonly the case in persistent UTIs (Sillankorva et al., 2011). While the assessment of bacteriophage activity in the context of pregnancy is lacking, we can appreciate the body of work regarding phage efficacy and selectivity in *in vitro*, animal and non-pregnant human models and move toward broadening the scope of this research.

*P. aeruginosa* represents another pathogen that causes UTIs from which isolation of MDR strains is becoming more frequent. Human examples of phage therapy often comprise case reports of persistent pathogens and instances of compassionate phage use. One such example includes a 67 year old woman administered a cocktail of six lytic phages in combination with antibiotic therapy to treat a UTI caused by *P. aeruginosa*. Antibiotic therapy alone had failed, however, combination of this with the phage cocktail resulted in eradication (Khawaldeh et al., 2011). Additionally, a clinical trial is currently underway to assess phage therapy in the context of UTI in transurethral resection of prostate patients (Leitner et al., 2017). While this trial is in men, the study design and context of UTIs make for a valuable addition to our current understanding of phage therapy in humans and will allow assessment of inter-patient variability which cannot be assessed from case studies. This represents significant progress toward gaining thorough and credible clinical trial data which will impact future phage therapeutics.

### Group B streptococcus

Intrapartum antibiotic prophylaxis for *S. agalactiae* or Group B Streptococcus (GBS) vaginal/rectal colonization is standard practice in many countries in an attempt to reduce neonatal morbidity and mortality associated with GBS disease. These prophylactic strategies vary from risk-based to culture-based screening and result in identified women receiving intravenous (IV) antibiotics a minimum of 4 h prior to delivery and then every 4 h during labor in an effort to eradicate the bacteria (Verani et al., 2010). With 10 to 30% of pregnant women colonized with GBS, the potential for antibiotic exposure is widespread (Le Doare and Heath, 2013). This is a prime example of a single species target in which phage therapy could excel.

Exploitation of the key components of virulent phage, such as the lysins that result in bacterial cell lysis, have been pursued as therapeutic options (Gaca and Gilmore, 2016). Bacteriophage lysins are responsible for the final stage of the lytic cycle which involves disruption of the bacterial host envelope and release of phage progeny and cell contents, inevitably destroying the host (Grundling et al., 2001). Exogenously, the lysin enzyme alone is often sufficient to lyse bacteria as it has direct contact with the peptidoglycan layer (Loeffler et al., 2001). This property has been targeted by researchers in an attempt to uncover novel therapeutic strategies. Pritchard and colleagues examined the bifunctional peptidoglycan lysin of GBS bacteriophage B30 and found a synergistic killing activity between the lysozyme and endopeptidase functions (Pritchard et al., 2004). This study involved *in vitro* testing and characterization of the lysin molecules, which they aimed to use as treatment for vaginal GBS in an effort to prevent GBS disease transmission. The following year Cheng et al. examined GBS lysin activity *in vivo* using a mouse model (Cheng et al., 2005). It was found that GBS phage lysin, PlyGBS, efficiently killed all tested GBS serotypes *in vitro*, and in the mouse model significantly reduced bacterial colonization in both the vagina and oropharynx with a single dose. This study outlines promising results for use of bacteriophage lysins for the eradication of GBS in the vagina in particular. Bacteriophage-derived lysin reviews have highlighted the activity against Gram positive organisms (Fischetti, 2008; Fenton et al., 2010), however activity against Gram negative organisms has also been observed (Lood et al., 2015) and discussed (Yang et al., 2014; Trudil, 2015). These studies, however, rely on static compounds of the phage as opposed to the whole phage which has the potential to increase its concentration *in vitro* exponentially, therefore, these lysins are likely to face similar issues to antibiotics in that dose, host metabolism/excretion and potential resistance (although this is likely to be less of an issue for lysins) may limit therapeutic use.

Various *in vitro* studies have assessed whole bacteriophages which possess activity against GBS strains; as yet only temperate phages have been identified in this organism. Studies such as that by Domelier and others characterized the prophages present upon induction of clinical GBS strains and identified these as belonging to the family *Siphoviridae* (Domelier et al., 2009). Similarly, Bai and colleagues characterized a temperate phage, JX01, induced from a bovine mastitis isolate and reported similar taxonomic observations (Bai et al., 2013). This study went on to further assess characteristics of the phage and to report that an absorption of 90% of phage particles occurred after 2.5 min incubation, followed by a 30 min latent period prior to release of a burst size of 20 virions per infected cell. Another group examined the activity of induced and modified *S. agalactiae* phages *in vitro* and reported on their activity against pathogenic strains from the environment (Brnakova et al., 2005). They also went further to analyse horizontal transfer of genetic elements. To our knowledge no virulent GBS bacteriophages have been reported in the literature to date; these would represent the ideal candidates for phage therapy. Theoretically speaking, such phages should certainly exist.

### Other antenatal and perinatal pathogens

Sepsis and meningitis resulting from bacterial infections are a significant burden in the neonatal population. A number of key pathogens have been targeted in phage therapy research and experimental trials of phage formulations tested. As is the case with the majority of phage studies, these have predominantly been *in vitro* or *in vivo* animal studies. Multi-drug resistant (MDR) *K. pneumoniae* is a major cause of neonatal disease and very difficult to treat. The action of a phage formulation active against MDR-*K. pneumoniae* within a septic mouse model has been described (Vinodkumar et al., 2005). These authors observed 100% rescue of the mice with immediate phage administration and 50% when treatment was delayed until mice were moribund. Considering therapeutic limitations faced by clinicians when treating MDR-infections, phage therapy could be a viable alternative. Similarly, a Polish study observed sterilization of cerebrospinal fluid of a MDR-*K. pneumoniae* infected child after oral phage administration (Stroj et al., 1999).

Regarding phage administration, Russian studies observed anti-phage antibody production in infants and children up to 15 years of age that was associated with time post-oral bacteriophage treatment (Pagava et al., 2011, 2012). Interestingly, patients less than 1 month of age did not produce antibodies until 30–60 days post-treatment and even then they were only detected in 20% of the participants. Children aged 1 month to 1 year of age showed antibody presence in 4–60% of cases 30–60 days post treatment and for children aged 1–15 the rate was 33.3–100%. These data suggest there may be an advantage to having naïve neonatal immune system when considering efficacy and immune clearance of phage preparations, however, a recent study by Roach and others observed conflicting results. Their findings suggest a synergy between the host immune system and phage lysis of target bacteria, termed “immunophage synergy.” This comes as a result of administering phage to treat *P. aeruginosa* infection in healthy compared to immunocompromised mice (Roach et al., 2017). In the antenatal and perinatal context the impact of limited immune defenses, such as those of a fetus or neonate, is a major consideration in terms of efficacy and further exploration into the specific immune components involved is warranted.

*L. monocytogenes* is a food-borne pathogen which causes sepsis, abortion and central nervous system dysfunction. While clinical cases are relatively rare, listeriosis is associated with high mortality, especially in immunocompromized patients such as pregnant women and neonates (Poulsen and Czuprynski, 2013). As a food-borne pathogen, prevention of infection at the production stage has seen bacteriophage approved for use as biocontrol agents by the Food and Drug Administration (FDA) (Lang, 2006). Klumpp and Loessner provide a thorough overview of *Listeria* spp. phages (Klumpp and Loessner, 2013) including the number of applications that have been achieved such as biocontrol on ready-to-eat foods (Guenther et al., 2009) and sprays for fresh fruit (Leverentz et al., 2004). Phage application as a preventative measure is currently more feasible than its use to treat human infection due to the intracellular nature of *L. monocytogenes* infection. Intracellular infection creates a barrier to phage therapy, however, studies are evaluating alternative phage access methods such as encapsulation in liposomes (Nieth et al., 2015) and avirulent co-infection models (Broxmeyer et al., 2002) in an attempt to overcome this problem. The issue of phage access during human infection with *L. monocytogenes* in particular, however, still remains a logistical challenge.

## Route of administration and transfer

**Figure 1:**
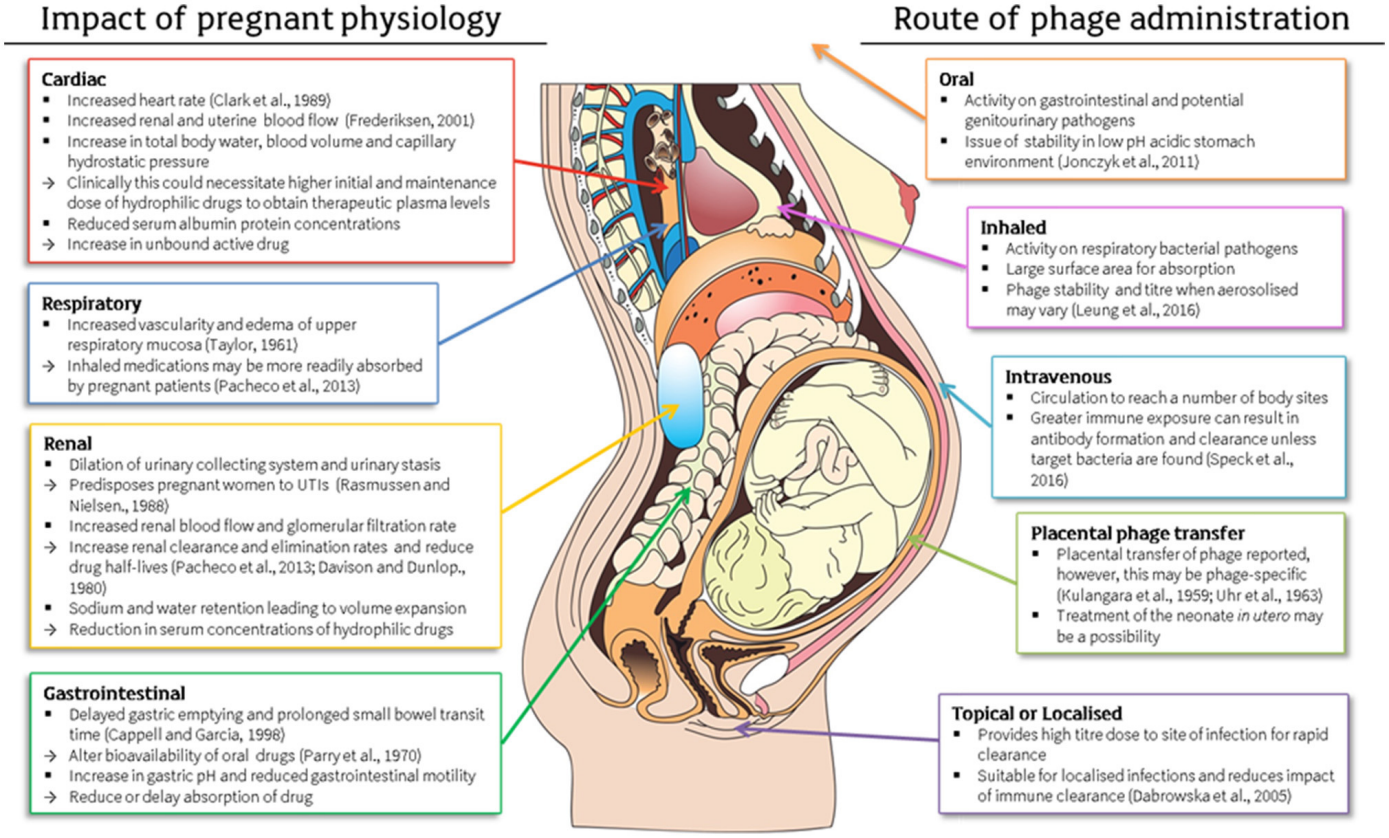
Physiological changes during pregnancy and their impact on drug pharmacokinetics and consideration of these factors in the application of antenatal phage therapy (Kulangara and Sellers, 1959; Taylor, 1961; Uhr et al., 1963; Parry et al., 1970; Davison and Dunlop, 1980; Rasmussen and Nielsen, 1988; Clark et al., 1989; Cappell and Garcia, 1998; Frederiksen, 2001; Dabrowska et al., 2005; Jonczyk et al., 2011; Pacheco et al., 2013; Costantine, 2014; Leung et al., 2016; Speck and Smithyman, 2016). This figure includes a licensed image obtained by the authors.

Availability of phages plays a key role in the efficacy of treatment. If just one phage can reach a bacterial cell of interest, then replication and release of exponential numbers of phage progeny will escalate the dose. Route of administration is dependent on the type and site of bacterial infection and this must be considered to ensure optimal results in terms of efficacy, distribution and clearance (Abedon, 2014). Once again, physiological differences during pregnancy can impact on therapeutic results through alterations in body systems (Costantine, 2014) including cardiac (Clark et al., 1989; Frederiksen, 2001; Pacheco et al., 2013), respiratory (Taylor, 1961; Pacheco et al., 2013), renal (Davison and Dunlop, 1980; Rasmussen and Nielsen, 1988; Pacheco et al., 2013) and gastrointestinal (Parry et al., 1970; Cappell and Garcia, 1998) systems for example (Figure 1).

### Intravenous phage administration

Systemic administration of phages by an IV route has the potential to treat multiple body sites, however a number of issues have been raised about the safety and efficacy of this application. A review by Speck and Smithyman highlighted and succinctly summarized these issues and previous successful IV phage applications (Speck and Smithyman, 2016). The main issues arising with IV use of phages can be summarized in the following categories; (i) clearance, (ii) adverse host response and (iii) host resistance; however, to some degree these are issues faced by all phage therapy routes.

The issue of clearance involves the rapid removal of phages from the bloodstream. The half-life of phages, however, has been described in the order of days as opposed to the hours observed for most antibiotics (Ochs et al., 1971). Further to this, Merril described selecting longer circulating phage variants for IV use (Merril et al., 1996). Equally, clearance is dependent on host bacteria presence, as the phage will persist only if they have access to their host bacteria to replicate in and subsequently lyse (Dubos et al., 1943).

Secondly, adverse host responses, such as shock, reported by early phage researchers (MacNeal, 1941) are likely due to the lack of purification of these initial preparations: current best practice involves extensive purification to ensure exclusion of bacterial components and endotoxins (Bonilla et al., 2016). The current state of bacteriophage preparation for IV use, however, has been refined significantly and has allowed successful IV administration and infection eradication as reported by numerous case studies in the US in the last year (Duplessis et al., 2017; Jennes et al., 2017; Schooley et al., 2017). Similarly, the effect of cell lysis and ensuing response to cell debris has been raised, however, this should be considered no different to the effects of bactericidal IV antibiotics which are regularly prescribed (Speck and Smithyman, 2016).

Lastly, the formation of phage-specific antibodies may act to clear the phage before they can exert an effect on the targeted pathogens. In response to this point we are reminded by the study of Matsuzaki et al. (2005) that phage kill extremely quickly and the time required for antibody formation may exceed what is needed to treat and resolve infection. Cases which involve prolonged treatment where antibodies are likely to form, however, may require administration of additional phages that do not have the same serological cross-reactivity (Abedon et al., 2011).

Considering the increased heart rate, uterine and renal blood flow observed in pregnancy the impact on IV phage administration may correlate with the findings observed for IV drug administration (Clark et al., 1989; Frederiksen, 2001). The increased cardiac output may require a greater dose to obtain initial plasma levels. Further to this, as phage are dose-independent in nature (replication upon bacterial interaction) the premise of adequate plasma levels is likely to be completely different to the way we view classical drugs. Attention should be focused on defining and refining such parameters in current phage studies of non-pregnant humans. Valuable information gained from each phage administration could be used to build individualized drug profiles for each phage which will likely influence future administration.

### Local–topical, oral or inhaled phage administration

The thorough review by Dabrowska and others outlined numerous studies that have revealed the extent of bacteriophage penetration in vertebrates (Dabrowska et al., 2005). This article explored the ability of bacteriophages to enter the circulation by non-IV means (oral, intraperitoneal, intramuscular and topical) and disseminate into internal organs and the central nervous system. It was suggested that phage persistence and concentration in particular organs strongly depends on the presence or absence of susceptible bacteria.

The local delivery of bacteriophages is most appropriate where a site-specific infection or colonization is occurring (Ryan et al., 2011). In pregnancy, intravaginal application could be ideal for delivery of phages specific to genitourinary tract pathogens. Similarly in neonates, skin based administration may act to protect the infant's fragile skin and prevent colonization by pathogens. Such applications may come in the form of phage-impregnated gels, creams and pessaries, all of which have retained phage activity in such formulations as previously demonstrated (Brown et al., 2017).

Topical applications are ideal as a high titre of bacteriophage can be delivered directly to the source of infection with minimal host immune interference. Common examples of this topical approach include skin and sinus infections by organisms such as *Staphylococcus aureus* (Seth et al., 2013; Pincus et al., 2015; Drilling et al., 2017). In antenatal terms, however, the major sources of infection relate to genitourinary pathogens (Cunnington et al., 2013). As GBS is commonly isolated from both the vagina and rectum (Dillon et al., 1982) this represents a target site for topical applications. Intravaginal delivery of bacteriophage lysin was previously demonstrated by Cheng and colleagues in a mouse model (Cheng et al., 2005). Rectal phage delivery also has high potential for success with an early study reporting detection of phage in the blood as early as 10 min after administration (Sechter et al., 1989).

Respiratory pathogens can be targeted through inhalation of phage preparations and respiratory physiology during pregnancy is likely to enhance the absorption of inhaled drugs (Pacheco et al., 2013) and therefore potentially phages. The methods of production of powders and aerosolized phage, however, can result in titre reduction (Leung et al., 2016). Inhaled phage studies are summarized in a review by Bodier-Montagutelli et al. (2017). Respiratory phage delivery, however, is more relevant in perinatal and pediatric populations of developing nations where bacterial pneumonia accounts for 15% of deaths in children under 5 years (Liu et al., 2015). Respiratory delivery of phage and its potential for enhanced absorption could be beneficial during pregnancy as another route of administration and depending on the resulting access to the fetus, treat *in utero* infection.

Oral delivery has been successful for gastrointestinal tract and some systemic infections, however, the low pH of the stomach is a big issue for phage stability (Jonczyk et al., 2011). This problem may be overcome using microencapsulation techniques (Ma et al., 2008; Colom et al., 2015). Other suggestions have included the neutralization of stomach acid prior to oral delivery, with one study suggesting yogurt may have a synergistic effect in overcoming this unfavorable acidic environment (Miedzybrodzki et al., 2017). Similarly, proton-pump inhibitors such as rabeprazole have been used in *Helicobacter pylori* treatment and act to inhibit gastric acid production (Sharara, 2005). These approaches may be useful for oral phage administration during pregnancy. The physiological changes cause an increase in gastric pH which is a potential benefit, depending on the pH stability range of the phage, and reduced gastric emptying resulting in extended exposure to these pH conditions. If able to transit successfully through to the bowel, prolonged small intestine passage is likely. In light of new data suggesting transcytosis of phage across gut epithelial cells being possible *in vitro*, depending on the downstream consequences of this process which remain unknown, this could be beneficial (Nguyen et al., 2017). This also raises questions regarding the potential for transcytosis across the placenta.

### Placental phage administration

When treating the mother and fetus, it is essential to have a thorough understanding of the placenta and the extent to which drug transfer occurs across this maternal/fetal barrier. Kulangara et al. (Kulangara and Sellers, 1959) examined the passage of coliphage and mycobacteriophages across the placental barrier of rats. Passage was achieved after injection into the uterine lumen as confirmed by presence of phage in the fetal fluids, however, maternal IV injection saw phage rarely reach the embryo. Conversely, transplacental transfer of bacteriophage was observed in a guinea pig model, where maternal IV bacteriophage administration resulted in the presence of bacteriophages in the fetal circulation (Uhr et al., 1963). Similarly, in a phage-display study an engineered T7 phage was injected into the tail vein of a pregnant rat and was subsequently recovered from fetal tissues 15 min after administration (Srivastava et al., 2004). The potential for virulent phage to target not only maternal infection, but also fetal, is extremely promising in the antenatal context. Mechanisms of this placental transfer of bacteriophage, however, are not well-understood at this stage and further studies are vital to progressing *in utero* treatment strategies.

## Issues to consider

Successful therapies during pregnancy rely on a number of intercalating factors. First, is the aim of the therapy to eradicate maternal or fetal infection, or could both be feasible? Placental transfer, fetal immune response or lack thereof, maternal immune response, dose, route of administration and pharmacokinetics all play key roles in therapeutic considerations. Some of the greatest challenges in phage therapy have been linked to immune inactivation by antibodies. As mentioned throughout this review, these are all aspects that can be overcome by altering the way we approach phage therapy. Extensive characterization, purification and preparation of cocktails of phage to act specifically but also broadly amongst different strains of the same target of interest are likely to permit widespread killing of the entire species without the adverse reactions described in early literature. In addition, having a thorough understanding of the infection that is to be treated is also extremely important, as in the case of *in utero* infections, for example, if secondary pathogens are also present (but in much smaller numbers), then targeted removal of the primary pathogen may simply pave the way for a new infection to begin. Treatment protocols need to factor this scenario in and have suitable complementary interventions on hand, in this case most likely antibiotics. The issue of the potential for host inflammatory response due to release of bacterial cell components following cell lysis is obviously of concern, especially in an antenatal context due to the nature of parturition, however, antibiotic interventions can also result in similar scenarios. Further research into this is required, particularly the potential for phage preparations to be co-administered with anti-inflammatory agents such as cytokine-suppressive anti-inflammatory drugs (Ng et al., 2015).

Phage therapy has had a difficult time integrating with western medicine and its use during pregnancy is largely unexplored. This review highlights how little we currently know about the safety of the most common drugs used in pregnancy, antibiotics, and the potential benefits that phage research into antenatal and perinatal infection and colonization could have in the future. Although extensive phage research has been carried out, the majority of this research has not been in the western world. In a clinical context, many have questioned the credibility of past studies due to the lack of randomized double-blind clinical trials in general (Slopek et al., 1985, 1987). In addition, a number of studies reporting the use of phage therapy in pregnant women and pediatric contexts are inaccessible or only available in Russian (Samsygina and Boni, 1984; Terekhina et al., 2008; Pagava et al., 2011). To overcome these current perceptions of phage therapy a basic science approach needs to be taken initially, whereby candidate clinical bacteriophages are thoroughly characterized (molecularly and taxonomically) and purified, and potential bacterial resistance monitored at the molecular level in the target bacteria and by analysis of mutations occurring in the phages. Such an approach would likely result in the formulation of well-characterized, robust, efficacious phage cocktails suitable for use in randomized clinical trials. It is essential that standardized protocols be developed, not only for uniformity but so that accurate comparisons of data can be made. Furthermore, the refinement of clinical practice can be largely influenced by case studies, and despite patient heterogeneity, similarities in phage pharmacokinetics may be observed which are informative nonetheless and should not impede standard care.

The ethical implications of phage therapy thus far have not been greatly explored, as many instances of human phage therapy have been for compassionate use as a final option. The potential for phage therapy to be beneficial for treatment of pregnant women and neonates is great when we reflect on the pathogens afflicting this population, however, there will no doubt be a number of ethical considerations that need to be addressed which are specific to these patients. Optimal healthcare is a priority and this will ultimately come down to the decision of the patient, who needs to be fully informed of the risks vs. benefits.

## Conclusion

The vulnerability of pregnant women and neonates as patients can make development and thorough testing of therapeutic agents difficult. This is evident in the little data available on antibiotic safety in pregnancy. Regardless of the ethical issues associated with this population, they remain at risk of infection and thus need access to safe, efficacious treatments. Antibiotics have numerous contraindications in pregnancy, have problems associated with resistance and new data suggests that antibiotic alteration of the vagina could influence the microbial seeding of the neonatal gut. Despite this, they still are certainly of use in pregnancy where an infection or potential infection of unknown etiology is in play, for example PROM. The potential for use of phage therapy is already fast accelerating with the emergence of antibiotic resistance, however, the targeted nature of this intervention would be especially beneficial in an antenatal and perinatal context where minimal disruption of the host microbiota is of importance. Through the numerous studies that have examined bacteriophages as therapeutic agents, we can see their vast and unique potential, especially in that their concentration and persistence is dependent on bacterial host prevalence and their site-specific localization appears to be somewhat independent of their route of administration. The potential for targeted treatment of infections in the mother and fetus is exciting and certainly warrants additional research into further development of antenatal *in vitro* and *in vivo* models of bacteriophage therapy to complement the many years of previous research in this field summarized within this review.

## Author contributions

LF conceived the review topic and focus, drafted the manuscript and figures, and approved the final version to be published. Both BC and MP contributed to the structure and content, critically revised the drafted manuscript and approved the final version to be published.

### Conflict of interest statement

The authors declare that the research was conducted in the absence of any commercial or financial relationships that could be construed as a potential conflict of interest.
